# Normalized performance and load data for the deepwind demonstrator in controlled conditions

**DOI:** 10.1016/j.dib.2016.07.029

**Published:** 2016-07-20

**Authors:** L. Battisti, E. Benini, A. Brighenti, M. Raciti Castelli, S. Dell’Anna, V. Dossena, G. Persico, U. Schmidt Paulsen, T.F. Pedersen

**Affiliations:** a Dipartimento di Ingegneria Civile Ambientale e Meccanica, Università di Trento, Italy; b Dipartimento di Energia, Politecnico di Milano, Italy; c Technical University of Denmark, Risø Campus, Roskilde, Denmark

**Keywords:** VAWT, DeepWind Project, Troposkien rotor, Skewed flow, Wind tunnel measurements, Wind turbine benchmark data

## Abstract

Performance and load normalized coefficients, deriving from an experimental campaign of measurements conducted at the large scale wind tunnel of the Politecnico di Milano (Italy), are presented with the aim of providing useful benchmark data for the validation of numerical codes. Rough data, derived from real scale measurements on a three-bladed Troposkien vertical-axis wind turbine, are manipulated in a convenient form to be easily compared with the typical outputs provided by simulation codes. The here proposed data complement and support the measurements already presented in “Wind Tunnel Testing of the DeepWind Demonstrator in Design and Tilted Operating Conditions” (Battisti et al., 2016) [1].

## Nomenclature

*A*: rotor swept area (m^2^)
*c*: blade chord length (m)
*C*_TX_: $\frac{{\text{T}}_{\text{X}}}{\text{0.5}\text{ρ}\text{A}{\text{V}}_{\text{∞}}^{\text{2}}}=$X-thrust coefficient (dimensionless)
*C*_TY_: $\frac{{\text{T}}_{\text{Y}}}{\text{0.5}\text{ρ}\text{A}{\text{V}}_{\text{∞}}^{\text{2}}}=$Y-thrust (dimensionless)
*C*_Q,aero_: $\frac{Q_{\mathrm{aero}}}{0.5\rho \mathrm{A R}V_{\infty }^{2}}=$torque coefficient (dimensionless)
*Q*_aero_: aerodynamic torque (Nm)
*R*: maximum turbine radius (m)
Re: $\frac{\text{ω}\text{R c}}{\text{ν}}=$chord Reynolds number (dimensionless)
*T*_X_: X-thrust (N)
*T*_Y_: Y-thrust (N)
TSR_eq_: $\frac{\omega R}{V_{\infty }}=$equatorial Tip Speed Ratio (dimensionless)
*V*_∞_: free stream wind speed (m/s)

**Greek**
*υ*: freestream air kinematic viscosity (m^2^/s)
*ρ*: freestream air density (kg/m^3^)
*Ω*: rotor angular speed (rpm)
*ω*: rotor angular speed (s^−^^1^)

**Specifications Table**

**Table:** 

Subject area	Physics
More specific subject area	Wind engineering
Type of data	Tables, graphs, figure
How data was acquired	Precision torquemeter, absolute encoder, 2 full strain gauge bridges
Data format	Filtered and analyzed
Experimental factors	Raw data are normalized using coefficients typically adopted in wind turbine engineering
Experimental features	Open jet wind tunnel and high precision test bench
Data source location	Trento, Italy
Data accessibility	All the data are presented in this article

**Value of the data**

**Fig. 1:**
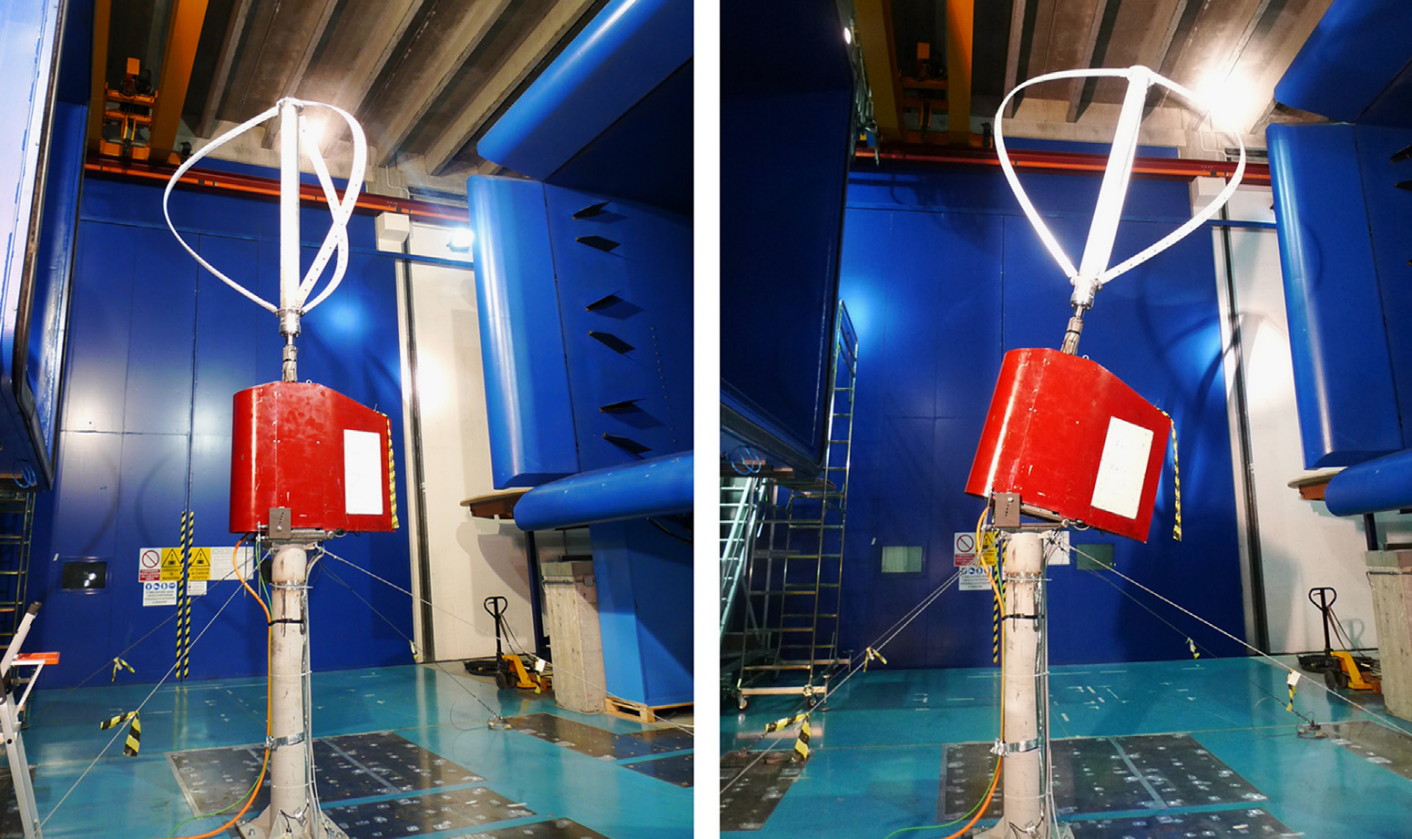
Side view of the Politecnico di Milano open chamber, showing also the tested rotor arrangement for both design (left) and tilted (right) operating conditions.
• Contrarily to open field testing, the here presented data allow numerical researchers to develop and validate enhanced engineering models on the basis of full-scale measurements conducted in an environment free from pronounced inflow anomalies.
• Great care is adopted in the description of the presented experimental data, in order to provide a useful benchmark for numerical simulations.

• The primary objective of the measurement campaign conducted on the DeepWind reduced scale demonstrator is to provide information needed to quantify the three-dimensional aerodynamic behavior of a Troposkien wind turbine in both design (i.e. upright) and tilted (up to 15° with respect to the vertical axis, as shown in Fig. 1) operating conditions to quantify the impact of the tilting angle.

### 1 Data

Aerodynamic raw data measured during the wind tunnel campaign were rotor torque (*Q*_aero_), rotor thrust (both in the longitudinal direction *T*_X_ and in the transversal one *T*_Y_), rotor rotational speed (ω) and wind tunnel speed ($V_{\infty }$). These data are here presented in a convenient form typically adopted in wind turbine engineering. As a matter of fact, to provide more insights on rotor behavior, aerodynamic torque (*C*_Q,aero_) and thrust (*C*_TX_ and *C*_TY_) coefficients are provided in Table 1, Table 2, Table 3, Table 4. as a function of the equatorial Tip Speed Ratio (TSR_eq_) computed at rotor equatorial diameter.

**Table 1:** Upright rotor at *Ω*=200 rpm.

**TSR_eq_**	***C***_**Q,aero**_	***C***_**TX**_	***C***_**TY**_
**[dimensionless]**	**[dimensionless]**	**[dimensionless]**	**[dimensionless]**
1.42	0.022	0.362	−0.116
1.52	0.025	0.378	−0.119
1.63	0.028	0.401	−0.124
1.78	0.030	0.426	−0.132
1.94	0.035	0.454	−0.135
2.12	0.047	0.496	−0.126
2.35	0.056	0.552	−0.127
2.63	0.064	0.612	−0.134
3.00	0.074	0.685	−0.150
3.28	0.077	0.715	−0.167
3.41	0.077	0.734	−0.171
3.53	0.078	0.763	−0.166
3.72	0.074	0.767	−0.166
3.89	0.067	0.764	−0.165
4.07	0.059	0.781	−0.170
4.26	0.046	0.798	−0.172
4.77	0.014	0.761	−0.195
5.34	−0.023	0.774	−0.217

**Table 2:** Upright rotor at *Ω*=300 rpm.

**TSR_eq_**	***C***_**Q,aero**_	***C***_**TX**_	***C***_**TY**_
**[dimensionless]**	**[dimensionless]**	**[dimensionless]**	**[dimensionless]**
2.12	0.063	0.505	−0.150
2.27	0.070	0.539	−0.154
2.45	0.078	0.579	−0.158
2.68	0.091	0.634	−0.160
2.92	0.101	0.692	−0.164
3.18	0.104	0.738	−0.167
3.38	0.102	0.763	−0.169
3.54	0.097	0.785	−0.165
3.76	0.088	0.798	−0.171
3.98	0.078	0.814	−0.166
4.26	0.062	0.817	−0.178
4.56	0.044	0.818	−0.181
4.91	0.024	0.806	−0.197
5.33	0.000	0.781	−0.205
6.39	−0.060	0.735	−0.246
8.17	−0.146	0.759	−0.338

**Table 3:** Tilted rotor at *Ω*=200 rpm.

**TSR_eq_**	***C***_**Q,aero**_	***C***_**TX**_	***C***_**TY**_
**[dimensionless]**	**[dimensionless]**	**[dimensionless]**	**[dimensionless]**
1.52	0.024	0.367	−0.123
1.64	0.027	0.388	−0.129
1.78	0.032	0.411	−0.132
1.94	0.035	0.441	−0.139
2.12	0.044	0.483	−0.134
2.36	0.053	0.527	−0.136
2.64	0.061	0.583	−0.147
3.03	0.069	0.664	−0.162
3.29	0.071	0.708	−0.175
3.54	0.065	0.733	−0.175
3.87	0.059	0.764	−0.185
4.21	0.041	0.766	−0.176
4.77	0.012	0.789	−0.203

**Table 4:** Tilted rotor at *Ω*=300 rpm.

**TSR_eq_**	***C***_**Q,aero**_	***C***_**TX**_	***C***_**TY**_
**[dimensionless]**	**[dimensionless]**	**[dimensionless]**	**[dimensionless]**
2.14	0.062	0.489	−0.146
2.29	0.068	0.521	−0.152
2.46	0.075	0.556	−0.156
2.67	0.082	0.602	−0.162
2.92	0.091	0.657	−0.165
3.20	0.094	0.709	−0.171
3.54	0.089	0.753	−0.173
4.01	0.059	0.783	−0.171
4.55	0.040	0.796	−0.181
5.28	−0.002	0.773	−0.201
6.25	−0.054	0.732	−0.240

**Fig. 2:**
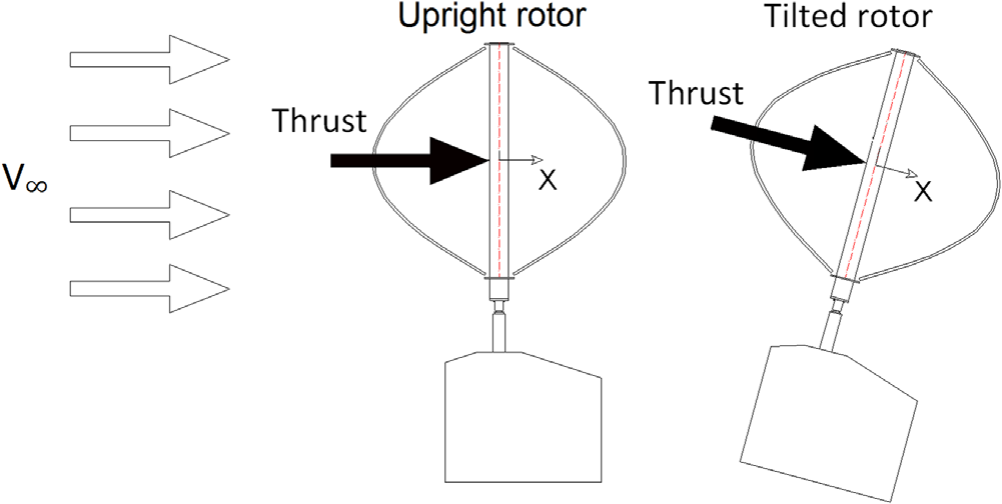
Scheme of the local coordinate system in the longitudinal direction.

**Fig. 3:**
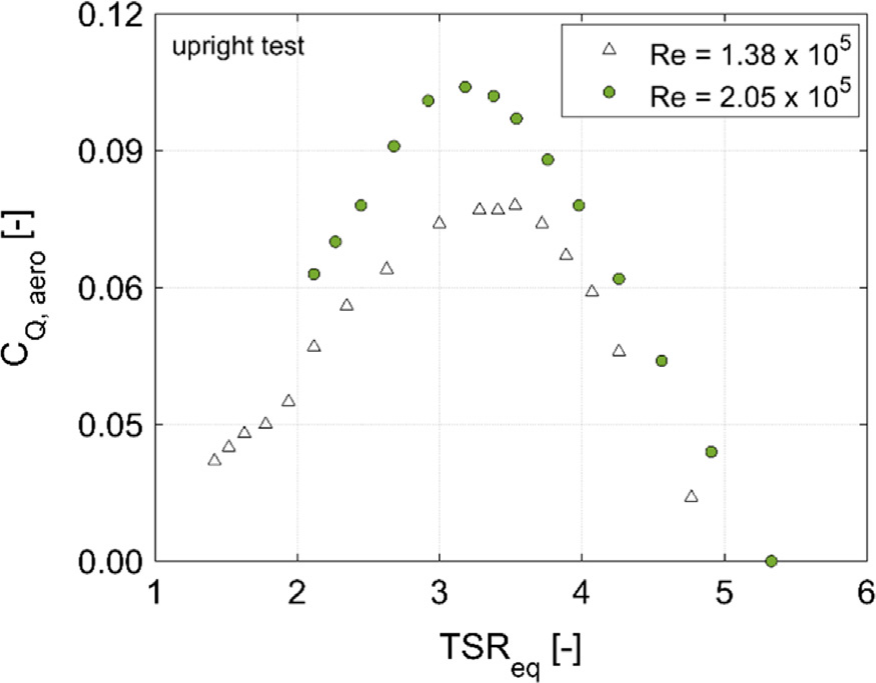
*C*_Q_ curves for the upright rotor at 200 rpm (Re=1.38×10^5^) and 300 rpm (Re=2.05×10^5^).

**Fig. 4:**
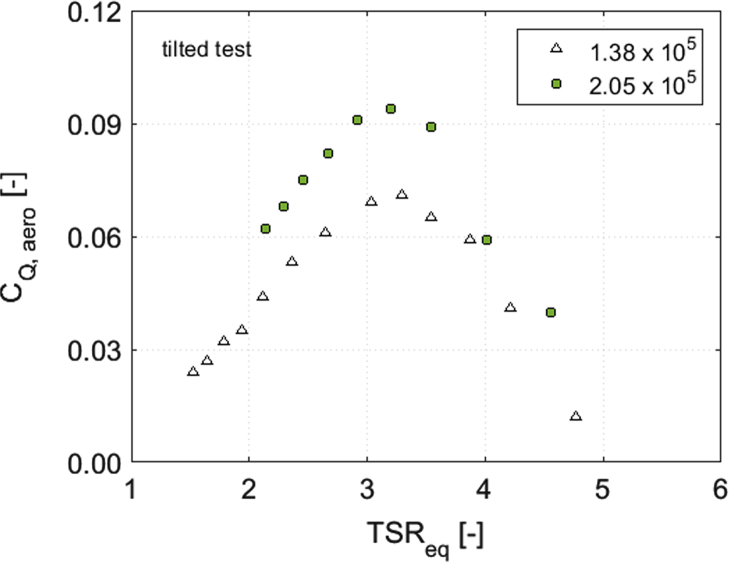
*C*_Q_ curves for the tilted rotor at 200 rpm (Re=1.38×10^5^) and 300 rpm (Re=2.05×10^5^).

**Fig. 5:**
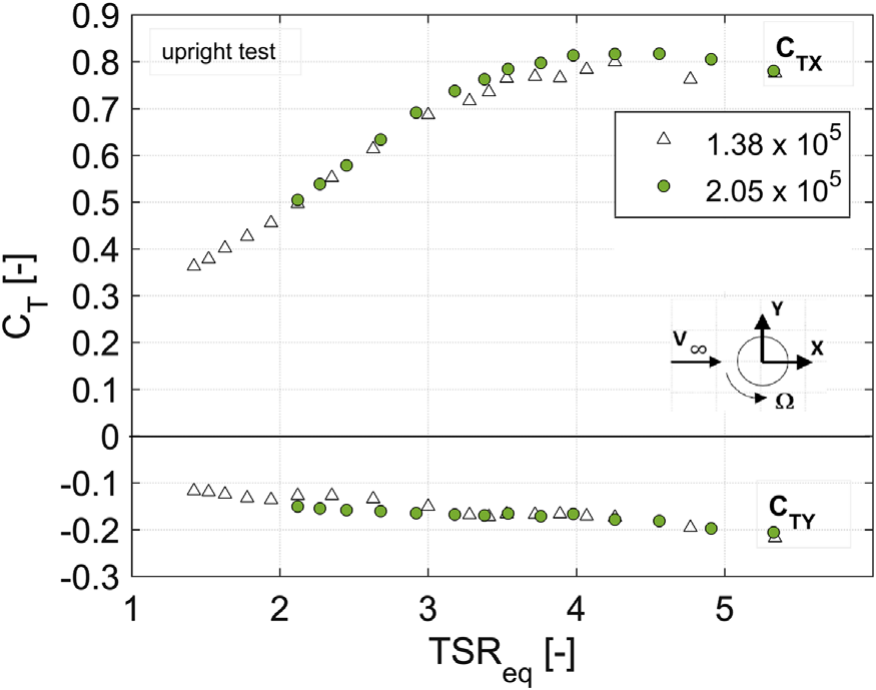
*C*_TX_ and *C*_TY_ curves for the upright rotor at 200 rpm (Re=1.38×10^5^) and 300 rpm (Re=2.05×10^5^).

**Fig. 6:**
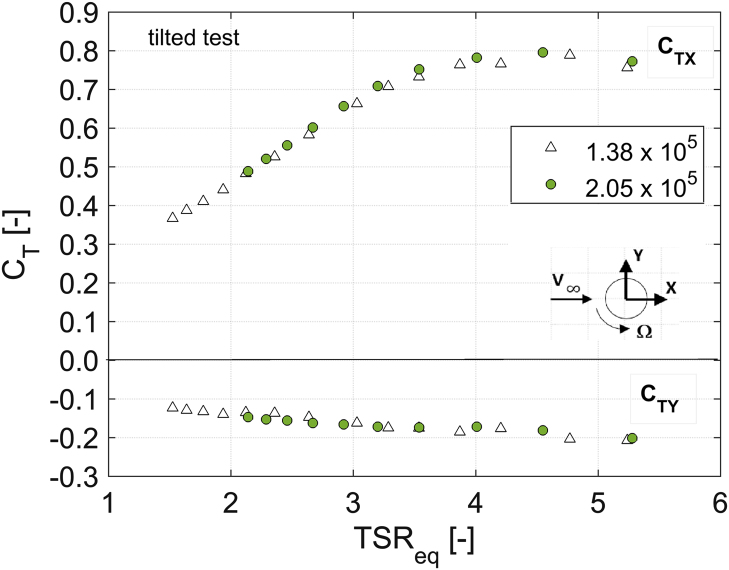
*C*_TX_ and *C*_TY_ curves for the tilted rotor at 200 rpm (Re=1.38×10^5^) and 300 rpm (Re=2.05×10^5^).

In order to disclose the influence of the blade Reynolds number (Re) on aerodynamic torque, power and thrust coefficients, Fig. 2, Fig. 3, Fig. 4, Fig. 5, Fig. 6 show a comparison between the data obtained at two rotor angular velocities, respectively 200 rpm (Re=1.38×10^5^) and 300 rpm (Re=2.05×10^5^).

### 2 Experimental design, materials and methods

The experimental campaign was conducted at the Politecnico di Milano (IT) large scale wind tunnel, characterized by a working section of 4.00 m width and 3.84 m height. The wind tunnel was operated in a “free jet” (open) configuration with a central section of 6.00 m length. Rotor torque and thrust measurements were taken using a high precision test bench, which was instrumented using a precision torquemeter (to provide rotor aerodynamic torque), an absolute encoder (to provide rotor angular velocity) and 2 full strain gauge bridges (to provide rotor aerodynamic thrusts in both the longitudinal direction and in the transversal one).

Both upright and 15° tilted rotor configurations were tested in the open jet wind tunnel, as schematized in Fig. 1, showing also the local coordinate system for the longitudinal (*X*) direction adopted during thrust measurements. It is worth observing that only the aerodynamic thrust is provided in all tables and graphs, i.e. no corrections have been introduced in order to avoid the rotor tower drag force. Furthermore, for tilted tests, the strain gauge offset has been recorded with tilted rotor: it was therefore possible to measure the wind thrust avoiding the component due to rotor weight bending moment.

See [1], [2] for more details regarding data acquisition and data processing techniques.
